# Chemical Profiling, Antioxidant, and Anti-Inflammatory Activities of *Hyoseris radiata* L., a Plant Used in the Phytoalimurgic Tradition

**DOI:** 10.3390/antiox13010111

**Published:** 2024-01-17

**Authors:** Maria Vitiello, Michela Pecoraro, Marinella De Leo, Fabiano Camangi, Valentina Parisi, Giuliana Donadio, Alessandra Braca, Silvia Franceschelli, Nunziatina De Tommasi

**Affiliations:** 1 Department of Pharmacy, University of Pisa, 56126 Pisa, Italy; maria.vitiello@phd.unipi.it (M.V.); marinella.deleo@unipi.it (M.D.L.); 2 Department of Pharmacy, University of Salerno, 84084 Fisciano, Italy; mipecoraro@unisa.it (M.P.); vparisi@unisa.it (V.P.); gdonadio@unisa.it (G.D.); sfranceschelli@unisa.it (S.F.); detommasi@unisa.it (N.D.T.); 3 Interdepartmental Research Center Nutrafood “Nutraceuticals and Food for Health”, University of Pisa, 56124 Pisa, Italy; 4 CISUP, Centre for Instrumentation Sharing, University of Pisa, 56127 Pisa, Italy; 5 CSRC, Crop Science Research Center, Sant’Anna School of Advanced Studies, 56127 Pisa, Italy; fabiano.camangi@santannapisa.it

**Keywords:** *Hyoseris radiata* L., LC-HR-ESI-MS, NMR, antioxidant, anti-inflammatory, phytoalimurgy

## Abstract

*Hyoseris radiata* L. (Asteraceae), known as “wild chicory”, is a perennial herbaceous plant native to the Mediterranean region, North Africa, and West Asia. Collected from the wild, the plant is largely used in Italy for culinary purposes and in popular medicine, so that it can be included in the list of phytoalimurgic plants. The present study aimed to investigate for the first time the plant’s chemical profile, through a combined UHPLC-HR-ESI-Orbitrap/MS and NMR approach, and its potential healthy properties, focusing on antioxidant and anti-inflammatory activities. The LC-MS/MS analysis and the isolation through chromatographic techniques of the plant’s hydroalcoholic extract allowed the authors to identify 48 compounds, including hydroxycinnamic acids, flavonoids, megastigmane glucosides, coumarins, and lignans, together with several unsaturated fatty acids. The quantitative analysis highlighted a relevant amount of flavonoids and hydroxycinnamic acids, with a total of 12.9 ± 0.4 mg/g DW. NMR-based chemical profiling revealed the presence of a good amount of amino acids and monosaccharides, and chicoric and chlorogenic acids as the most representative polyphenols. Finally, the antioxidant and anti-inflammatory activities of *H. radiata* were investigated through cell-free and cell-based assays, showing a good antioxidant potential for the plant extract and a significant reduction in COX-2 expression.

## 1. Introduction

Wild food species constitute an important component of the diet of many people across the world. The Food and Agriculture Organization (FAO) reported that around 20% of the population in the EU appreciates the consumption of wild foods, including wild edible plants (WEPs) [1]. The importance of WEPs has increased, especially in times of war and famine, when they served as a crucial resource for poor communities, hence being designated as “famine foods” [2]. Although their consumption has been decreasing over time, wild plants have recently been rediscovered as a food and included both in the local and haute cuisine of many countries, also due to their beneficial properties.

**Figure 1:**
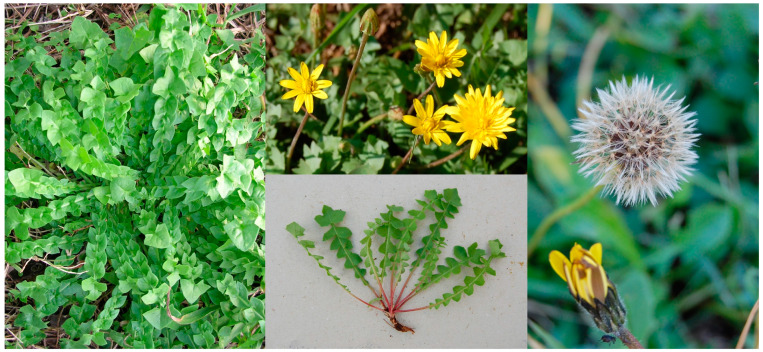
*Hyoseris radiata* L. (Asteraceae family).

*Hyoseris radiata* L. (Figure 1), commonly known as “wild chicory”, is a perennial herbaceous plant (hemicryptophyte rosulate) belonging to the Asteraceae family, native to the Mediterranean region, in particular Europe (France, Greece, Italy, Malta, Spain, and ex-Yugoslavia), North Africa (Algeria, Canary Islands, Morocco, and Tunisia), and West Asia (Turkey) [3,4]. It grows at altitudes of up to 1000 m a.s.l. and it can be found in pastures, in uncultivated fields, on roadsides, and between the stones of dry walls. The plant is 1–40 cm tall, the stems are erect, aphyllous, glabrous or hispid, with a tap root. The leaves (2–2.5 × 10–14 mm) are collected in the basal rosette and are petiolates, oblanceolate, and pinnate in shape, with ovate lobes (6–9 on each side), often runcinate, imbricate, and dentate. The receptacle is flat without scales. The flowers are all ligulate, bright yellow and collected in flower heads (3–4.5 cm) with involucral bracts in two rows: the outer series are short and oblong bracts (4–5 mm), the inner series are long and linear bracts (10–15 mm). The fruits are brown-colored achenes (8–11 mm) and have a silky pappus. The plant flowers throughout the year. The karyotype is 2*n* = 16 [5].

The traditional use of this “wild chicory“is well known in Italy [6], in particular, in the central–southern areas [7,8,9] and on the major islands [10,11,12], while in northern Italy, it is limited to some regions of the north-west [13], such as Liguria [14]. The young leaves are eaten raw in salads or boiled, alone or together with other wild herbs, and drizzled with olive oil and lemon or vinegar, browned in oil or stewed. The tender basal rosettes are also used to prepared omelets, savory pies, vegetable potages, and other traditional dishes such as “Gattafin di Levanto” in Liguria (fried pasta containing a mixture of vegetables) [6] and mixed soups such as “Preboggion or Prebuggiun“, again in Liguria [15], the “Zuppa delle 18 erbe“ from Barbagia in Sardinia [11], and the “Cucina massese/carrarese“ in Tuscany [16]. Furthermore, in Sardinia, young stems are eaten fresh as snacks by children due to their sweetish taste [11]; in Gargano (Puglia), the inflorescences are used raw as a snack, with bread or in salads [17]; in Calabria, the leaves are preserved in olive oil to be eaten as a vegetable side dish [18]. This plant is also used for medicinal purposes [19]. In Italy, popular medicine recommends an infusion of its leaves to drink as a diuretic [20,21], as a blood depurative [22] or as a litholithic against kidney stones [11]. Another common use is to boil the leaves as an intestinal depurative and in order to avoid excessive constipation. Moreover, the raw leaves are a food integrator for rabbits and sheep [14].

Despite its large use in popular medicine and traditional culinary purposes, *H. radiata* has been poorly studied to date. Previous old phytochemical studies reported the isolation of some acetylenes from the roots and lupeyl acetate and taraxasteryl acetate from the aerial parts [23], as well antioxidant activity, spectrophotometrically determined in extracts from the leaves, accounting for a good content of phenols and flavonoids [18,22].

Since WEPs such as *H. radiata* could show excellent organoleptic characteristics, different culinary uses, flavors, low maintenance to common diseases, and good capability of growing in wild and ruderal areas, their valorization is highly recommended. Thus, due to the lack of a complete phytochemical characterization of this popularly consumed plant and its potential in providing health benefits, the aim of this study was the investigation for the first time of *H. radiata’s* chemical components in term of bioactive agents and nutrients. Then, cell-free and cell-based assays were used to investigate the antioxidants and anti-inflammatory activities of the plant extract for a future application of this species for healthy and nutritional purposes and to valorize it as a possible future stress-tolerant crop. This work could be included in the tentative strategies for the economic valorization of WEPs, strongly encouraged to preserve native wild species and to generate economic returns to local communities.

## 2. Materials and Methods

### 2.1. Chemicals and Apparatus

All analytical-grade solvents were purchased from VWR (Milano, Italy). MeOH, H_2_O, and HCOOH used for ultra-high-performance chromatography (UHPLC) were purchased from Merck KGaA (Merck KGaA, Darmstadt, Germany). Kaempferol 3-*O*-rutinoside (≥98% purity) and chicoric acid (≥95% purity) were purchased from Extrasynthese (Extrasynthese, Genay, France) and Merck Life Science (Merck Life Science s.r.l., Milano, Italy), respectively. Alanine (≥98% purity), asparagine (≥98% purity), proline (≥98% purity), arginine (≥98% purity), threonine (≥99% purity), valine (≥98% purity), sucrose (≥98% purity), fructose (≥98% purity), KH_2_PO_4,_ D_2_O (99.9 atom % D), D_2_O (99.9 atom % D) containing 0.75 wt. % of 3-(trimethylsilyl)propionic-2,2,3,3-*d*_4_ acid sodium salt (TSP) for NMR analysis were purchased from Merck Life Science (Merck Life Science s.r.l., Milano, Italy). Thin-layer chromatography (TLC) was carried out using silica gel 60 F_254_ (0.20 mm thickness) plates, *n*-BuOH-CH_3_COOH-H_2_O (60:15:25) as an eluent, and cerium sulphate as spray reagent (Merck Life Science s.r.l., Milano, Italy). 2,2′-Azino-bis-3-ethylbenzthiazoline-6-sulphonic acid (ABTS) and 1,1-diphenyl-2-picrylhydrazyl (DPPH) were purchased from Merck Life Science (Merk Life Science s.r.l., Milano, Italy). Gel filtration chromatography was carried out over Sephadex LH-20 (40–70 µm) column using a peristaltic pump (Pharmacia, Uppsala, Sweden). Reverse-phase high-performance liquid chromatography (RP-HPLC) was conducted using a Shimadzu LC-8A series pumping system equipped with a Shimadzu RID-10A refractive index detector (Shimadzu, Milano, Italy) and a C18 μ-Bondapak column (30 cm × 7.8 mm, 10 µm particle size; Waters, Milano, Italy). UHPLC coupled to high-resolution mass spectrometry (HR-MS) was carried out with a Vanquish Flex Binary pump coupled to a HR-MS Q Exactive Plus Orbitrap-based FT-MS equipped with an electrospray ionization (ESI) source and Xcalibur 4.1 software (Thermo Fisher Scientific Inc., Bremen, Germany). The separation was performed using C-18 Kinetex^®^ Biphenyl column (100 × 2.1 mm, 2.6 µm particle size) provided with a Security Guard^TM^ Ultra Cartridge (Phenomenex, Bologna, Italy). Mono- and bidimensional NMR spectra for chemical characterization of pure compounds were acquired in methanol-*d_4_* on Bruker DRX-400 spectrometer (Bruker BioSpinGmBH, Rheinstetten, Germany). Data were processed with Topspin version 3.2. The NMR experiments for quali-quantitative profiling of the extract were acquired in D_2_O on Bruker Avance 600 spectrometer (Bruker BioSpinGmBH, Rheinstetten, Germany) equipped with a 5 mm probe operating at 298 K and SampleJet autosampler.

### 2.2. Plant Material and Extract Preparation

Leaves of *Hyoseris radiata* L. (2434.5 g) were collected in Antignano (Livorno, Italy) in March 2022 and identified by Dr. Fabiano Camangi. A voucher specimen (N° PI064810) was deposited at the Herbarium Horti Botanici Pisani (Pisa, Italy).

The leaves were dried in a ventilated oven at 39 °C, obtaining 315.0 g of dried material, that was powdered using a MF 10 basic Microfine grinder drive (IKA-Werke, Staufen, Germany) with a sieve of 0.25 mm hole size, and subjected to a single ultrasound-assisted extraction (LBS2 bath, Falc Instruments s.r.l., Treviglio, Italy) for 15 min at 20 °C and 59 kH frequency, utilizing EtOH 80% *v*/*v* (solid:liquid ratio of 1:10 g/mL). After removing the solvent under vacuum by using Rotavapor^®^ (Buchi, Milano, Italy) 59.2 g of dried extract was obtained.

### 2.3. Extract Fractionation and Isolation of Pure Components

A part of the hydroalcoholic extract (9.5 g) was dissolved in 25 mL of MeOH and centrifugated for 5 min at 2710× *g*, then the supernatant was chromatographed on Sephadex LH-20 column, using MeOH as eluent at a flow rate of 1 mL/min. A total of 117 fractions of 17 mL each were collected and grouped into 11 major fractions (A-K) based on TLC results. Fraction C (200.0 mg) was subjected to RP-HPLC eluting with MeOH-H_2_O (3:7, *v*/*v*) to give compounds **23** (3.6 mg, *t*_R_ 13.3 min), **8** (2.1 mg, *t*_R_ 19.8 min), and **10** (3.2 mg, *t*_R_ 25.7 min). Fraction D (186.7 mg) was purified by RP-HPLC with MeOH-H_2_O (35:75, *v*/*v*) as eluent to obtain compound **15** (0.1 mg, *t*_R_ 58.4 min). Fraction E (42.6 mg) was submitted to RP-HPLC with MeOH−H_2_O (3:7, *v*/*v*) to yield compound **7** (0.1 mg, *t*_R_ 13.1 min). From fraction F (238.8 mg) eluted with MeOH−H_2_O (3:7, *v*/*v*), compounds **9** (1.3 mg, *t*_R_ 16.0 min) and **12** (2.2 mg, *t*_R_ 23.1 min) were isolated. Fractions H and I were combined into one fraction (60.0 mg) that was then separated by RP-HPLC eluting with MeOH−H_2_O (4:6, *v*/*v*) to give compound **20** (2.1 mg, *t*_R_ 47.1 min). Fraction J (25.4 mg) was submitted to RP-HPLC eluting with MeOH−H_2_O (65:35, *v*/*v*) to yield compounds **2** (2.4 mg, *t*_R_ 8.4 min) and **25** (1.4 mg, *t*_R_ 12.6 min). Finally, fraction K was eluted as a pure compound (**31**) from Sephadex column (7.1 mg).

### 2.4. Chemical Characterization of the Extract by LC-HR-Orbitrap/ESI-MS

The hydroalcoholic extract (10.0 mg) was dissolved in 5 mL of MeOH (2 mg/mL final concentration), centrifuged for 5 min at 2710× *g,* and 5 µL of supernatant was directly injected into the LC-MS/MS system. The operation was repeated three times to obtain three solutions. The analysis was carried out using a mobile phase composed of a mixture of HCOOH in H_2_O 0.1% *v*/*v* (solvent A) and HCOOH in MeOH 0.1% *v*/*v* (solvent B), and a linear gradient of 5 to 100% of solvent B for 26 min at a flow rate of 0.5 mL/min. The HR-MS spectra were acquired both in positive and negative ion mode, within a *m*/*z* scan range of 135–2000, using the ionization parameters as previously reported [24]. The MS operated in full MS/MS scan (70,000 resolution, maximum injection time 220 ms) and data dependent (17,500 resolution, maximum injection time 60 ms). The column and autosampler temperature were maintained at 35 and 4 °C, respectively. The identification of all compounds was tentatively defined by their accurate measured mass, and the comparison of their elution order, and both full and fragmentation mass spectra, with data reported in the literature. Isolated compounds were used as a reference to confirm their identification in the LC-MS profile. For the quantitative analysis of the major chemical constituents, chicoric acid and kaempferol 3-*O*-rutinoside were used as external standards for phenolic acids and flavonoids, respectively. Stock methanol solutions (1 mg/mL) were first prepared and then diluted by serial dilution to obtain solutions in triplicate at the range of 0.05–0.00156 mg/mL for both standards. The calibration curves were constructed, plotting two variables, concentrations, and areas obtained by MS peak integration, which were related by the following linear simple correlation: *R*^2^ = 0.9992 for chicoric acid and *R*^2^ = 0.9978 for kaempferol 3-*O*-rutinoside. Data were processed by Microsoft^®^ Office Excel (Microsoft 365 version 2312), and the amounts of components were finally expressed as mg/g ± standard deviation (SD) of dried weight (DW) of leaves.

### 2.5. NMR Quali-Quantitative Profiling of H. radiata Extract

Briefly, three aliquots of 7 mg each of dried extract were dissolved in 0.7 mL KH_2_PO_4_ buffer in D_2_O (pH 6.0), containing 0.01% 3TSP for a final concentration of 10 mg/mL. After centrifugation at 13,000 rpm for 10 min, the clear supernatants (600 μL) were transferred into NMR tubes for further analysis. ^1^H NMR spectra were recorded using a NOESY (noesygppr1d) pulse sequence with water signal suppression. Acquisition parameters were 8000 Hz (13.3 ppm) spectral width, 4 dummy, and 64 scans, a recycle delay of 4 s, mixing time 0.01 s, and a fixed value for receiver gain for all samples. NMR spectra processing (baseline correction, ppm calibration, variable-sized bucketing) and metabolite quantification were performed using NMRProcFlow (INRAE UMR 1332 BFP, Bordeaux Metabolomics Facility, Villenave d’Ornon, France) [25,26]. Alanine, valine, asparagine, sucrose, glucose, and galactose were selected for quantification due to the presence of isolate signals in NMR spectrum. Alanine and sucrose were used as external standards for the quantification of amino acids and sugars, respectively. The calibration curve of alanine was made in a concentration range from 10 to 500 µg/mL (y = 42618x − 8843.5 *R*^2^ = 1). The calibration curve of sucrose was made in a concentration range from 20 to 10,000 µg/mL (y = 3629.6x + 34,583 *R*^2^ = 0.9999). Data were exported into a spreadsheet workbook using the “qHNMR” template and processed with Microsoft^®^ Office Excel (Microsoft 365 version 2312), and the amounts of components were finally expressed as mg/g ± SD of leaf DW.

### 2.6. Radical-Scavenging Activity Assays

To evaluate the antioxidant power of the hydroalcoholic extract of *H. radiata,* two most common radical-scavenging assays using ABTS (2,2′-azino-bis(3-ethylbenzothiazoline-6-sulfonic acid) and DPPH (2,20-diphenyl-1-picrylhydrazyl radical) radicals were performed [27,28]. Samples were compared to known concentrations of Trolox standards, a water-soluble analog of tocopherol (vitamin E), which is a very strong antioxidant that is commonly used to measure antioxidant capacity. Different Trolox mM concentrations were incubated in the presence of the DPPH and ABTS radicals, and calibration curves were constructed with Trolox concentrations; the standard curve was linear between 0.025 and 1 mM Trolox. For both assays, extract was diluted between 500 and 2000-fold. For DPPH assay, the reaction was allowed to proceed for 30 min in the dark at room temperature, and then the decrease in absorbance at 515 nm was measured. For ABTS assay, after the addition of 100 µL of extract solutions to 100 µL of ABTS^•+^ solution, the absorbance reading was taken at 30 °C for 10 min after initial mixing and its absorbance was measured at 734 nm. Following that, the appropriate dilution factor was applied to calculate the millimolar TE of the extract at 10 mg/mL. All solutions were used on the day of preparation and all determinations were carried out in triplicate. The absorbance of the sample was recorded using spectrophotometer instrument.

### 2.7. Cell Culture

Human cell lines of alveolar adenocarcinoma (A549) and epidermal keratinocyte (HaCaT) were grown in Dulbecco’s Mixture F-12 Ham and Dulbecco’s modified Eagle’s medium, respectively, containing high glucose supplemented with 10% fetal bovine serum, 100 U/mL each of penicillin and streptomycin, in a humidified atmosphere of 5% CO_2_ at 37 °C. Cells were used at less than 80% of confluence.

### 2.8. Cell Viability Assay

Cell viability was analyzed via MTT (3-[4,5-dimetiltiazol-2,5-diphenyl-2H-tetrazolium bromide]) assay [29]. Briefly, cells (3.5 × 10^3^/well) were grown in 96-well plates and, after 24 h, were treated with fresh medium alone or containing different extract concentrations (100, 50, 25, and 10 μg/mL), for 24 or 48 h. Staurosporin 0.2 µM was used as a positive control. After treatment, MTT (5 mg/mL) was added to each well and plates were incubated for further 3 h, allowing salt formazan to crystallize. Then, salt formazan was solubilized with 100 μL of DMSO, and absorbance at 550 nm for each well was evaluated with a Multiskan Spectrum Thermo Electron Corporation Reader. Cell vitality was determined as % vitality = 100 × (OD treated/OD DMSO).

### 2.9. Cellular Antioxidant Activity Assay

*H. radiata* extract’s antioxidant activity was analyzed via a cytofluorimetric assay using 2′,7′-dichlorofluorescein diacetate (H_2_DCF-DA, D6883, Sigma-Aldrich, St. Louis, MO, USA). Intracellular esterases hydrolyze H_2_DCF-DA, a non-polar molecule that readily diffuses into cells, removing the acetate groups to make the molecule polar and impermeable. Intracellular Reacting Oxygen Species (ROS) rapidly oxidize H_2_DCF-DA into highly fluorescent DCF detectable and quantifiable via flow cytometry (excitation wavelength: 504 nm, emission wavelength: 529 nm). Shortly, A549 and HaCaT cells were seeded with a density of 3.0 × 10^4^ cells/well in 12-well plates. Based on the data obtained from the viability assay, 50 and 25 µg/mL of plant extract was added for 1 h, and the mixture was then co-exposed to lipopolisaccaride (LPS) from *Escherichia coli* (0.1 µg/mL) [30]. After 24 and 48 h, cells were collected, washed twice with phosphate buffered saline (PBS), and incubated in PBS containing H_2_DCF-DA (10 µM) at 37 °C. After 45 min, cell fluorescence was measured via flow cytometry and analyzed with Cell Quest software version 4.1 [31].

### 2.10. Measurement of COX-2 Expression

COX-2 (sc-19999, Dallas, TX, USA) expression was detected via fluorescence-activated cell sorting (FACSscan; Becton–Dickinson, BD Biosciences, San Jose, CA, USA). Cells were cultured in a 12-well plate (3 × 10^4^ cells/well) and, after 24 h of adhesion, treated as for the antioxidant activity assay. After, cells were collected and incubated with a fixing solution (4% formaldehyde, 2% fetal bovine serum (FBS), and sodium azide 0.1% in PBS) for 20 min, and then permeabilized with a buffer containing 4% formaldehyde, 2% FBS, Triton X-0.1%, and PBS in presence of 0.1% sodium azide for 30 min. Subsequently anti-COX-2 was added, and anti-goat Texas-Red (T6390, Waltham, MA, USA) was used as a secondary antibody. After washing, cells were fixed and detected via flow cytofluorometry and analyzed with Cell Quest software. Data were depicted as positive cells percentage [31].

### 2.11. Data Analysis

Data evaluations and statistical analysis were reported with commercially available software GraphPad Prism8 (GraphPad Software Inc., version 8.0.2, San Diego CA, USA). Results are represented as mean ± standard error of the mean (SEM) values of at least three different experiments performed in technical triplicate. Statistical analyses were obtained thanks to non-parametric Mann–Whitney U test. Differences were considered significant if *p*-values were from <0.01 to 0.05.

## 3. Results

### 3.1. Isolation and Identification of Pure Components

The hydroalcoholic extract of *H. radiata* leaves was subjected to fractionation via different chromatographic techniques, leading to the isolation of eight pure components. All compounds were structurally characterized by comparison of 1D and 2D NMR and MS spectra with the literature data, leading to identification of two hydroxycinnamic acids as chlorogenic acid (**9**) [32] and chicoric acid (**12**) [33]; three megastigmane glucosides as debiloside C (**23**) [34], alangionoside E (**8**), and plucheoside B (**10**) [35]; a lignan as secoisolaricinesinol *O*-β-d-glucopyranoside (**15**) [36]; a coumarin as cichoriin (**7**) [37]; three flavonoids as luteolin 7-*O*-rutinoside (**20**), kaempferol 3-*O*-glucoside (**25**), and luteolin (**31**) [38]; and a purine nucleoside as adenosine (**2**) [39]. The purity of all compounds met the criteria of >95% purity as inferred using UHPLC and NMR analyses.

### 3.2. Chemical Fingerprimt and Amount of Components via LC-MS/MS Analysis

#### 3.2.1. Qualitative Analysis

**Figure 2:**
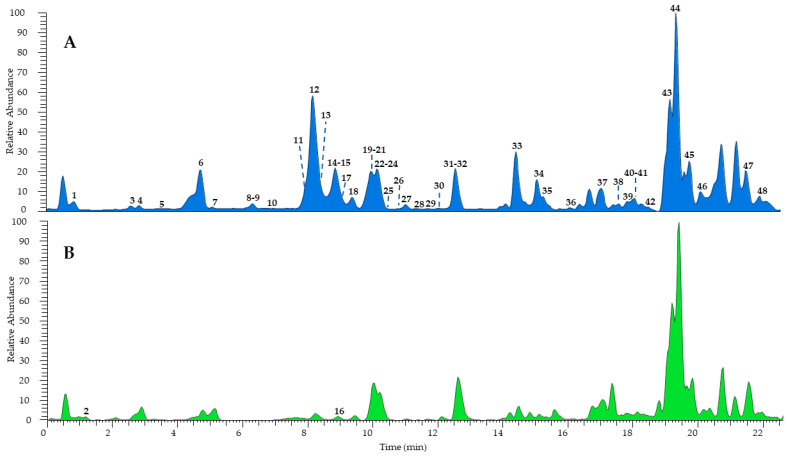
UHPLC-HR-ESI-Orbitrap/MS profiles of *H. radiata* leaf extract recorded in negative (**A**) and positive ionization mode (**B**). Peak data are shown in Table 1.

The combination of different techniques can be useful for a comprehensive metabolomic study of complex natural mixtures. Therefore, the hydroalcoholic extract of *H. radiata* leaves was investigated firstly via UHPLC-HR-ESI-Orbitrap/MS, leading to the identification of a great number of components, also present in traces, compared to the fractionation process, which failed in some cases due to very close retention times and the poor quantity of several samples. According to MS data, retention time, and comparison with isolated compounds and the literature data [24,26], the LC-MS profile (Figure 2) was characterized by 48 compounds, mostly phenols belonging to different subclasses (Table 1). According to the results obtained via extract fractionation, hydroxycinnamic acids, flavonoids (kaempferol, luteolin, quercetin, and apigenin derivatives), megastigmane glucosides, coumarins, and lignans were identified, together with a great number of unsaturated fatty acids.

Among phenols, eleven hydroxycinnamic acids were detected and tentatively identified as derivatives of caffeic, ferulic, and quinic acids. The most represented compounds were chlorogenic acid (**9**, [M − H]^−^ at *m*/*z* 353.0879) and chicoric acid (**12**, [M − H]^−^ at *m*/*z* 473.0717), confirmed by injections of pure isolated compounds. The peak at *m*/*z* 311.0403 ([M − H]^−^) displayed deprotonated tartaric acid at *m*/*z* 149.01 and caffeic acid at *m*/*z* 179.03 in the MS*^2^* experiments; thus, it was assigned as caftaric acid (**3**). Compound **26** was tentatively identified as caffeoylferuloyltartaric acid showing a molecular deprotonated ion [M − H]^−^ at *m*/*z* 487.0873 and product ions [M – H − 162]^−^ at *m*/*z* 325.05 and [M – H – 162 − 132]^−^ at *m*/*z* 193.05, due to the loss of a caffeoyl and subsequently of a tartaroyl unit, respectively. Similarly, compound **32** showed the same molecular deprotonated ion and fragmentation pattern as **26** but a base ion peak at *m*/*z* 163.02 ([M – 162 − 162]^−^) due to the loss of both caffeoyl and feruloyl residues; therefore, it was identified as a caffeoylferuloyltartaric acid isomer. Furthermore, eleven compounds were tentatively identified as flavonoids. In detail, compound **20** showed a molecular deprotonated ion at *m*/*z* 593.1505 and product ions at *m*/*z* 447.09 ([M – H − 146]^−^) and 285.04 ([M – H – 146 − 162]^−^), generated by the loss of a rhamnose and a rutinose unit, respectively, leading the authors to identify the molecule as luteolin 7-*O*-rutinoside, also confirmed by the injection of the pure isolated compound. Compounds **21** and **30** were annotated as luteolin or kaempferol hexoside isomers based on the deprotonated aglycone at *m*/*z* 285.04 in the MS*^2^* due to the loss of a hexose residue. Compound **13** ([M − H]^−^ at *m*/*z* 609.1454) was identified as kaempferol or luteolin *O*-dihexoside due the sequential loss of two hexose residues in the fragmentation process and the product ion at *m*/*z* 285.04. On the other hand, compound **17** showed the same deprotonated molecular ion as **13** but a base ion peak at *m*/*z* 300.02, allowing its characterization as rutin. The presence of the product ion at *m*/*z* 269.04 in the fragmentation pathway of compounds **28** and **29** allowed their annotation as apigenin hexoside and apigenin uronide, respectively. Compound **25** ([M − H]^−^ at *m*/*z* 447.0926) and compound **31** ([M − H]^−^ at *m*/*z* 285.0406) were assigned as kaempferol 3-*O*-glucoside and luteolin, respectively, and their structure was confirmed by comparison with the isolated standards. The loss of uronic moiety ([M – H − 176]^−^) and the presence of the base ion peak ion at *m*/*z* 285.04 in the MS*^2^* of compound **22** allowed its tentative identification as kaempferol or luteolin *O*-uronide. Moreover, other minor compounds were detected: megastigmane glucosides (**8**, **10**, and **23**), coumarin (**7**), lignans (**15** and **24**), monoterpene (**16**), and unsaturated fatty acids (**33**–**48**), mainly hydroxylated and with a C_18_ chain. Compounds **2** and **16** were recorded only in positive ionization mode as protonated molecular ions ([M + H]^+^ at *m*/*z* 268.1032 and 197.1167, respectively); thus, ESI+ ion product ions were reported.

#### 3.2.2. Quantitative Analysis

LC-MS quantitative analysis (Table 2) highlighted a relevant amount of flavonoids and hydroxycinnamic acids in *H. radiata* hydroalcoholic extract. Luteolin/kaempferol hexoside and luteolin/kaempferol uronide (2.31 ± 0.06 and 2.60 ± 0.02 mg/g DW ± SD, respectively) were among the most abundant flavonoids in the extract, followed by luteolin, present in the form of aglycone in a comparable amount. Among phenolic acids, chicoric acid (**12**) was the most abundant (2.62 ± 0.04 mg/g DW ± SD), followed by chlorogenic acid (0.936 ± 0.04 mg/g DW ± SD), while compound **26**, annotated as caffeoylferuloyl tartaric acid, was the less abundant component (0.0482 ± 0.01 mg/g DW ± SD).

### 3.3. NMR-Based Metabolomic Profiling

#### 3.3.1. Qualitative Analysis

**Figure 3:**
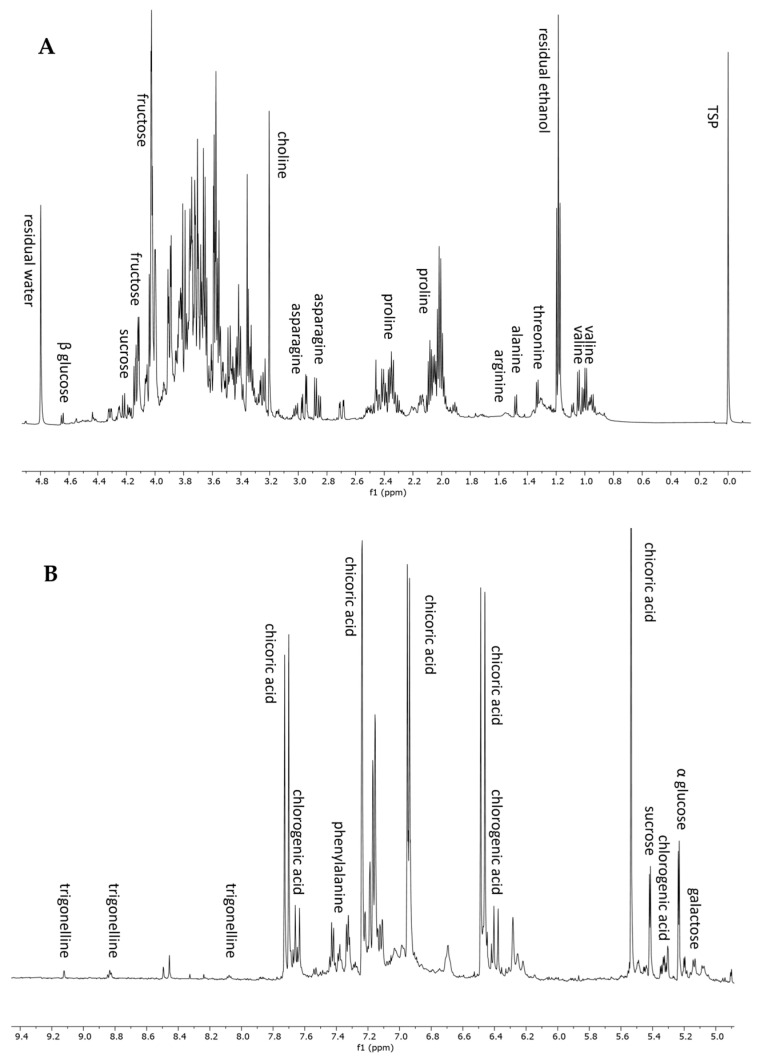
^1^H NMR spectrum from 0 to 4.9 ppm (**A**) and from 4.9 to 9.4 ppm (**B**) of *H. radiata* leaf extract and metabolites annotation.

To complete the metabolomic profiling of *H. radiata* hydroalcoholic extract, an NMR-based approach was also applied. ^1^H NMR analysis is a rapid and reproducible tool for the detection of primary metabolites and highly concentrated constituents. The ^1^H NMR spectrum of the plant extract showed the presence of seven amino acids, five sugars, two polyphenols, and an alkaloid. In detail, the aliphatic region, from 0 to 3 ppm (Figure 3A), displayed resonances corresponding to valine, threonine, alanine, arginine, proline, and asparagine. The annotation of these metabolites was also confirmed using standard compounds. In the sugar region, from 3 to 5.5 ppm, it was possible to recognize sucrose, glucose (α and β), and galactose, thanks to the chemical shift of anomeric protons (Table 3). The aromatic region of the spectrum (Figure 3B) was dominated by the resonance of chicoric acid, one of the most abundant polyphenols according to LC-MS analysis, and chlorogenic acid. The ^1^H-NMR spectrum resonances were first assigned according to the literature data [26,40], as well as public databases (HMDB, BMRB), and then confirmed through 2D NMR experiments (^1^H-^13^C HSQC and ^1^H-^13^C HMBC) and the use of pure standard compounds. Table 3 lists the chemical shift data for the annotated metabolites, with their level of identification according to Sumner et al., 2007 [41].

#### 3.3.2. Quantitative Analysis

^1^H NMR-based quantitative analysis of selected compounds, displaying well-resolved resonances, was performed. Among amino acids, alanine, valine, and asparagine were selected for quantitative analysis, and the results showed a content of 0.36 ± 0.01, 0.77 ± 0.01, and 1.05 ± 0.06 mg/g DW ± SD, respectively, in *H. radiata* dried leaves. Among selected sugars, sucrose is the most abundant in the dried leaves (4.17 ± 0.15 mg/g DW ± SD), followed by glucose (2.87 ± 0.16 mg/g DW ± SD) and galactose (0.29 ± 0.02 mg/g DW ± SD).

### 3.4. Antioxidant Activity

#### 3.4.1. Cell-Free Assays

The antioxidant activity assay of *H. radiata* extract was performed with ABTS and DPPH tests. These assays showed the antioxidant capacity of the extract estimated in terms of Trolox Equivalent Antioxidant Capacity (TEAC). TEAC was expressed as mM TE. The extract was tested at 10 mg/mL, and the TE was calculated. The results showed a radical-scavenging potential of 3.13 ± 0.04 mM and 2.43 ± 0.05 mM TE for ABTS and DPPH assays, respectively, suggesting good antioxidant activity for the extract, if compared to quercetin (TE 7.52 mM), a flavonoid with potent antioxidant activity [42].

#### 3.4.2. In-Cell Assay

**Figure 4:**
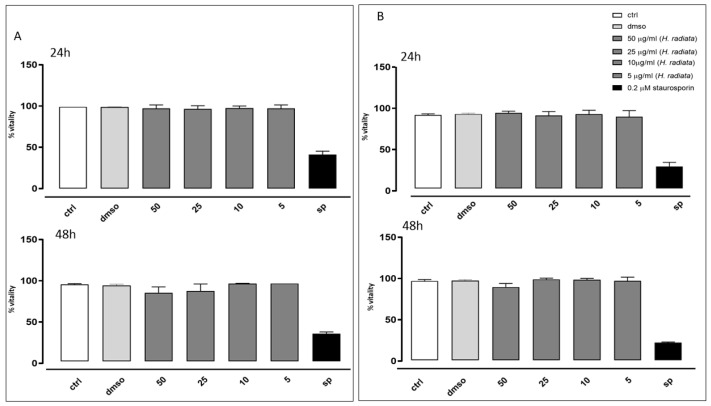
Cell vitality of A549 (**A**) and HaCaT (**B**) cells was calculated as % vitality = [100 × (OD treated/OD control)]. Results were analyzed by non-parametric Mann–Whitney U test.

The antioxidant activity of *H. radiata* extract was also evaluated in cells. Firstly, the extract was assayed to evaluate its effect on cell viability. Results obtained in our experimental models showed that different concentrations (50, 25, 10, and 5 μg/mL) of the extract did not induce a reduction in cell viability on the analyzed cell lines (Figure 4), both at 24 and 48 h of treatment. Furthermore, based on these data, 50 and 25 μg/mL were the selected concentrations for the subsequent in-cell antioxidant assay.

**Figure 5:**
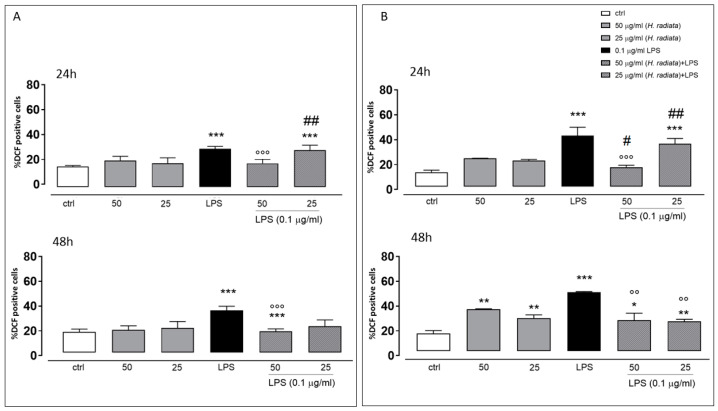
ROS formation was evaluated through the probe 2′.7′-dichlorofluorescein diacetate (H_2_DCF-DA) in A549 (**A**) and HaCaT (**B**) cells. *H. radiata* leaf extracts (50 and 25 µg/mL) were added for 1 h and then for further 24 h exposed to LPS from *Escherichia coli* (0.1 μg/mL). ROS production is expressed as mean ± SEM of the percentage of DCF-positive cells of at least three independent experiments, each performed in triplicate. Data were analyzed with Mann–Whitney U test. * *p* < 0.05, ** *p* < 0.005, and *** *p* < 0.001 versus untreated cells; °° *p* < 0.005, and °°° *p* < 0.001 versus LPS; # *p* < 0.05, and ## *p* < 0.005 versus treated cells at the same experimental conditions.

To test the antioxidant effect of the plant extract on ROS release in LPS-stimulated cells, the intracellular ROS via incubating cells with the *H. radiata* hydroalcoholic extract (50 and 25 µg/mL) 1 h before and simultaneously with LPS (0.1 µg/mL) was evaluated. After 24 and 48 h, LPS induced a significant ROS increase. The extract at the higher concentration tested, in the presence of LPS, significantly (*p* < 0.0001) reduced ROS release in respect to cells treated with LPS alone, at both experimental times (Figure 5).

### 3.5. Anti-Inflammatory Activity

**Figure 6:**
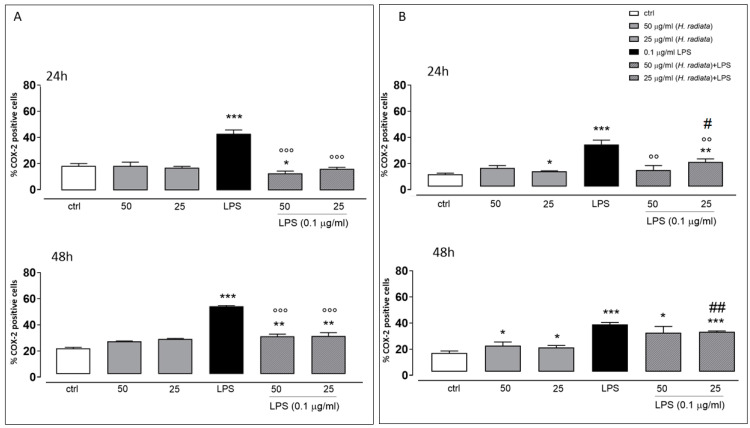
Panel (**A**) and (**B**) show COX-2 expression detected via flow cytometry analysis in A549 and HaCaT cells, respectively. Data are expressed as mean ± SEM of percentage of caspase 4-positive cells from at least three independent experiments, each performed in duplicate. Data were analyzed with Mann-Whitney U test. * *p* < 0.05, ** *p* < 0.005, and *** *p* < 0.001 versus untreated cells; °° *p* < 0.005, and °°° *p* < 0.001 versus LPS; # *p* < 0.05, and ## *p* < 0.005 versus treated cells at the same experimental conditions.

To investigate if *H. radiata* hydroalcoholic extract influences the inflammatory pathway, the COX-2 expression in A549 and HaCaT cells was analyzed, incubating cells with the extract (50 and 25 µg/mL) 1 h before and simultaneously with LPS (0.1 µg/mL). After 24 and 48 h, LPS induced a significant (*p* < 0.0001) COX-2 expression increase. The extract at the higher concentration tested, in the presence of LPS, significantly reduced COX-2 expression if compared to cells treated with LPS alone, at both experimental times (Figure 6).

## 4. Discussion

*H. radiata* is an unexplored WEP, despite its widespread use in traditional culinary recipes and popular medicine. In our study, a combined LC-MS and NMR approach was applied for the first time to analyze the specialized and primary small metabolites content of this WEP. The LC-MS/MS analysis and the isolation and characterization through chromatographic and spectroscopic techniques allowed the authors to identify 48 compounds, mainly including polyphenol derivatives, together with megastigmane glucosides and several unsaturated fatty acids. The quantitative analysis highlighted a relevant amount of bioactive molecules such as hydroxycinnamic acids and flavonoids, with a total of 12.9 ± 0.4 mg/g DW, chicoric acid and luteolin derivatives being the most abundant components. The NMR-based metabolomic profiling revealed the presence of a good quantity of amino acids, monosaccharides, and chicoric acid as the most representative polyphenol. A comparison of *H. radiata’s* specialized metabolites composition with that of correlated species, such as chicory (*Cichorium intybus* L.) and escarole cultivars (*Cichorium endivia* L.), revealed some similarities regarding the presence of phenolic compounds and flavonoids [26,43]. Anyway, cultivated chicory is mainly characterized by the presence in the roots of sesquiterpene lactones that were also revealed in the leaves of escarole cultivars. The chicoric acid content of *H. radiata* (2.62 ± 0.04 mg/g DW) was detected to be in the range of the one reported for cultivated chicory leaves (0.87 to 6.14 mg/g) [44]. The nutritional composition, although the comparison is not easy due to the variability among the different chicory and escarole cultivars, depending also on the cultivation technique, mineral fertilization, and/or soil salinization, is comparable and could justify further studies to enhance *H. radiata’s* exploitation. In fact, the edible leaves of *H. radiata* reported a high content of phenolic acids, flavonoids, and unsaturated fatty acids, conferring to the species important nutraceutical value.

This interesting profile in terms of nutritional and bioactive molecules prompted the investigation of the potential health properties of *H. radiata* leaves. In the last decades, it became evident that the antioxidant and anti-inflammatory properties of plant extracts and molecules seems to exert a beneficial effect upon several chronic inflammatory diseases and impairments. In particular, the presence of unsaturated fatty acid and polyphenols is very important, due to their effect on the inflammation process and on the cardiovascular system. Hence, to evaluate if the *H. radiata* polyphenols-rich extract possessed antioxidant and anti-inflammatory activities, cell-free and cell-based assays were implemented. Inflammation, a pathological condition considered as the cause of aging processes, generally induces oxidative stress, reducing antioxidant capacity. Consequently, the consumption of food and phytoalimurgic plants exerting both antioxidant and anti-inflammatory activities is desirable.

The preliminary evaluation of the potential cytotoxicity of this extract showed no activity at the tested concentrations. Since the ABTS and TEAC assays showed an interesting antioxidant activity for the plant extract, the antioxidant effect on ROS release in LPS-stimulated cell was also evaluated. Overproduction of ROS, due to LPS induction, leads to pathological effects caused by deleterious oxidative changes in cellular lipids, proteins, and DNA, as well as the formation of inflammatory proteins. Furthermore, it is known that the pro-inflammatory pathway is also induced by increased ROS levels [31]. Flow cytometric analysis using the fluorescent probe DCHF-DA showed that the *H. radiata* extract significantly reduced cytosolic ROS, confirming the results obtained by in vitro assays. The antioxidant activity, studied through cell-free and cell-based assay, exhibited by this extract could be linked to high phenolic content, confirming a correlation between these constituents and antioxidant activity. Indeed, in this study, the leaf extract of *H. radiata* showed a high amount of phenolic compounds, chicoric acid and luteolin derivatives being the most abundant components. Since the antioxidant activity of these molecules is well known and established in the literature [45,46,47], the total antioxidant activity exerted by *H. radiata* extract could be attributed to these phytocomplex components.

It has been reported that LPS-induced COX-2 expression is reduced by antioxidant treatment [48]. Therefore, the effect of *H. radiata* extract on COX-2 expression was evaluated. COX-2 is easily induced by LPS, supporting the establishment of an inflammatory condition, and represents the predominant protein at sites of inflammation. *H. radiata* extract was able to significantly reduce the expression of this protein, thus reducing the ongoing inflammatory state.

## 5. Conclusions

In popular tradition, wild plants, berries, and mushrooms have always represented an undisputed food resource, sometimes even an economical one, especially during times of war and famine. Among these species, the so called “phytoalimurgic plants” or WEPs, edible species of wild flora, are still used in the traditional folklore, although in a clear phase of decline due to the well-known and modified lifestyles and socio-cultural values imposed by progress and globalization. To counteract the loss of these traditional habits and enhance their value, our group was involved in a project aimed at biodiversity recovery and the exploitation of Mediterranean wild plants and fruits [26,49,50].

The results presented herein for the first time demonstrated that the WEP *H. radiata*, being a good source of polyphenols, amino acids, sugars, and polyunsaturated fatty acids could be used as a vegetable or in food preparations. According with these valuable data, a future aim will be to collect the species from different habitats and regions, to compare their chemical content, confirming these data, and to select the best area for its future cultivation and exploitation as new stress-tolerant crop. The agri-food sector could benefit from its exploitation and introduction to market, since this WEP has antioxidant and anti-inflammatory properties which corroborate its health and nutritional potential.

## Figures and Tables

**Table 1:** UHPLC-HR-ESI-Orbitrap/MS data of compounds detected in the leaf extract of *H. radiata*.

N. ^a^	Compound	*t*_R_ (min)	[M − H]^−^	Product Ions ^b^	Formula	Error
Hydroxycinnamic acids						
**3**	Caftaric acid	2.6	311.0403	292.89, 274.88, 179.03, **149.01**	C_13_H_12_O_9_	−1.46
**5**	Caffeic acid hexoside	3.6	341.0872	**179.03**, 135.05	C_15_H_18_O_9_	−1.79
**6**	Chlorogenic acid (isomer I) ^c^	4.7	353.0879	**191.05**, 173.04	C_16_H_18_O_9_	+0.25
**9**	Chlorogenic acid (isomer II)	6.3	353.0879	**191.05**, 173.04	C_16_H_18_O_9_	+0.25
**11**	Feruloylquinic acid	7.9	367.1027	**191.06**, 173.04	C_17_H_20_O_9_	−2.04
**12**	Chicoric acid (isomer I) ^c^	8.2	473.0717	311.04, 293.03, 179.03, **149.01**	C_22_H_18_O_12_	−1.80
**14**	Chicoric acid (isomer II)	8.9	473.0717	311.04, 293.03, 179.03, **149.01**	C_22_H_18_O_12_	−1.80
**19**	Dicaffeoylquinic acid (isomer I)	9.9	515.1186	353.09, **191.06**, 173.04	C_25_H_24_O_12_	−1.73
**26**	Caffeoylferuloyltartaric acid (isomer I)	10.8	487.0873	325.05, 293.03, **193.05**, 179.03, 112.99	C_23_H_20_O_12_	−1.83
**27**	Dicaffeoylquinic acid (isomer II)	11.1	515.1186	**353.09**, 191.06, 173.04	C_25_H_24_O_12_	−1.73
**32**	Caffeoylferuloyltartaric acid (isomer II)	12.7	487.0873	325.05, 293.03, 193.05, 179.03, **163.02**, 112.99	C_23_H_20_O_12_	−1.83
Flavonoids						
**13**	Kaempferol/Luteolin *O*-dihexoside	8.4	609.1454	447.09, **285.04**	C_27_H_30_O_16_	−1.15
**17**	Rutin	9.2	609.1456	**300.02**	C_27_H_30_O_16_	−0.84
**18**	Quercetin hexoside	9.4	463.0877	**300.02**	C_21_H_20_O_12_	−1.08
**20**	Luteolin 7-*O*-rutinoside ^c^	9.9	593.1505	447.09, **285.04**	C_27_H_30_O_15_	−1.16
**21**	Luteolin/Kaempferol hexoside (isomer I)	9.9	447.0926	**285.04**	C_21_H_20_O_11_	−1.52
**22**	Kaempferol/Luteolin *O*-uronide	10.2	461.0720	**285.04**	C_21_H_18_O_12_	−1.17
**25**	Kaempferol 3-*O*-glucoside ^c^	10.5	447.0926	**285.04**	C_21_H_20_O_11_	−1.52
**28**	Apigenin hexoside	11.4	431.0977	**269.04**	C_21_H_20_O_10_	−1.55
**29**	Apigenin uronide	11.7	445.0770	**269.04**	C_21_H_18_O_11_	−1.41
**30**	Luteolin/Kaempferol hexoside (isomer II)	12.1	447.0926	**285.04**	C_21_H_20_O_11_	−1.52
**31**	Luteolin ^c^	12.5	285.0406		C_15_H_10_O_6_	+1.96
Megastigmane glucosides						
**8**	Alangionoside E ^c^	6.2	433.2069 [M + HCOO]^−^	387.20, 165.04	C_19_H_32_O_8_	−2.25
**10**	Plucheoside B ^c^	6.8	433.2069 [M + HCOO]^−^	**387.20**, 161.04, 113.02, 101.02	C_19_H_32_O_8_	−2.25
**23**	Debiloside C ^c^	10.2	405.1762	243.12, **225.12**, 181.23, 163.12	C_18_H_30_O_10_	−1.04
Cumarins						
**7**	Cichoriin ^c^	5.3	339.0715	**177.02**	C_15_H_16_O_9_	−1.77
Lignans						
**15**	Secoisolariciresinol glucoside ^c^	8.9	523.2173	361.17	C_26_H_36_O_11_	−2.25
**24**	Secoisolariciresinol glucoside isomer	10.2	569.2231 [M + HCOO]^−^	361.17	C_26_H_36_O_11_	−1.51
Monoterpenes						
**16**	Loliolide ^d^	9.0	197.1167 [M + H]^+^	179.11, 161.10, 135.12	C_11_H_16_O_3_	−2.64
Primary metabolites						
**1**	Hexosylglutamic acid	0.8	290.0879	290.09, 272.07, 254.07, 230.07, 200.06, **128.03**	C_11_H_17_O_8_N	−0.83
**2**	Adenosine ^c,d^	1.1	268.1032 [M + H]^+^	136.06	C_10_H_13_N_5_O_4_	−3.10
**4**	Tryptophan	2.8	203.0819	186.05, 159.09, 142.06, **116.05**	C_11_H_12_O_2_N_2_	−3.45
Fatty acids						
**33**	Trihydroxyoctadecadienoic acid	14.5	327.2170	**327.21**, 309.21, 291.20, 229.14, 211.13	C_18_H_32_O_5_	−2.14
**34**	Trihydroxyoctadecenoic acid	15.1	329.2329	**329.23**, 311.22, 293.21	C_18_H_34_O_5_	−1.37
**35**	Dodecenoic acid	15.5	227.1287	209.12, **183.34**	C_12_H_20_O_4_	−0.79
**36**	Dihydroxyhexadecanoic acid	15.8	287.2223	269.21	C_16_H_32_O_4_	−1.67
**37**	Dioxooctadecadienoic acid isomer I	17.1	307.1910	289.18, 260.96, **235.14**	C_18_H_28_O_4_	−1.56
**38**	Dioxooctadecadienoic acid isomer II	17.6	307.1910	289.18, 260.96, **235.14**	C_18_H_28_O_4_	−1.56
**39**	Dihydroxyoctadecatrienoic acid	17.8	309.2066	**309.21**, 291.20, 273.18	C_18_H_30_O_4_	−1.71
**40**	Dihydroxyoctadecenoic acid	18.1	313.2380	**313.24**, 295.22, 277.22	C_18_H_34_O_4_	−1.37
**41**	Dioxooctadecatrienoic acid	18.2	305.1752	287.16, 249.15, **135.08**	C_18_H_26_O_4_	−2.06
**42**	Dihydroxyoctadecadienoic acid	18.5	311.2223	**311.22**, 293.21, 274.88	C_18_H_32_O_4_	−1.54
**43**	Hydroxyoctadecatrienoic acid isomer I	19.1	293.2116	**293.21**, 275.20, 249.16, 183.14	C_18_H_30_O_3_	−2.11
**44**	Hydroxyoctadecatrienoic acid isomer II	19.3	293.2116	**293.21**, 275.20, 249.16, 183.14	C_18_H_30_O_3_	−2.11
**45**	Hydroxyoctadecadienoic acid	19.7	295.2272	**295.23**, 277.22, 259.21, 195.14	C_18_H_32_O_3_	−2.23
**46**	Oxooctadecatrienoic acid	20.1	291.1989	273.18	C_18_H_28_O_3_	−0.69
**47**	Octadecatrienoic acid	21.5	277.2168	259.21	C_18_H_30_O_2_	−1.80
**48**	Octadecadienoic acid	21.8	279.2323	261.22	C_18_H_32_O_2_	−1.97

^a^ Peaks are listed by their elution order. ^b^ Base peak is shown in bold. ^c^ Confirmed by injection of reference standards. ^d^ MS data are recorded in positive ion mode.

**Table 2:** Amount (mg/g DW ± SD) of major flavonoids and hydroxycinnamic acids detected in *H. radiata* leaves.

Peak	Compound	mg/g DW ± SD
*Flavonoids*		
**18**	Quercetin hexoside	0.695 ± 0.1
**20**	Luteolin 7-*O*-rutinoside	0.576 ± 0.06
**21**	Luteolin/Kaempferol hexoside (isomer I)	2.31 ± 0.06
**22**	Luteolin/Kaempferol uronide	2.60 ± 0.02
**30**	Luteolin/Kaempferol hexoside (isomer II)	0.120 ± 0.01
**31**	Luteolin	2.15 ± 0.03
*Hydroxycinnamic acids*		
**3**	Caftaric acid	0.126 ± 0.004
**5**	Caffeic acid hexoside	0.102 ± 0.007
**6 + 9**	Chlorogenic acid	0.936 ± 0.04
**12**	Chicoric acid	2.62 ± 0.04
**19 + 27**	Dicaffeoylquinic acid	0.628 ± 0.04
**26**	Caffeoylferuloyltartaric acid	0.0482 ± 0.01
	*Total flavonoids*	8.45 ± 0.3
	*Total hydroxycinnamic acids*	4.46 ± 0.1
	*Total*	12.9 ± 0.4

DW: dry weight; SD: standard deviation.

**Table 3:** Metabolites and ^1^H chemical shifts identified by 600 MHz 1D ^1^H-NMR for *H. radiata* leaf extract.

Compound	Chemical Shift (ppm) Multiplicity (*J* in Hz)	Identification Confirmation	MSI Status ^a^
Valine	0.99 d (*J* = 7.4), 1.04 d (*J* = 7.4)	Spike, HSQC	1
Threonine	1.33 (*J* = 6.8)	Spike, HSQC	1
Alanine	1.48 d (*J* = 7.2)	Spike, HSQC	1
Arginine	1.54 m	Spike, HSQC	1
Proline	2.03 m, 2.35 m	Spike, HSQC	1
Asparagine	2.85 dd (*J* = 16.7; 7.3), 2.96 dd (*J* = 16.2; 4.0)	Spike, HSQC	1
Choline	3.20 s	HSQC	2
α-Glucose	5.16 d (*J* = 4.40)	HSQC	2
β-Glucose	4.54 d (*J* = 7.95)	HSQC	2
Sucrose	5.40 d (*J* = 3.80)	Spike, HSQC	1
Fructose	4.01 m	Spike, HSQC	1
α-Galactose	5.20 d (*J* = 3.90)	HSQC	2
Chlorogenic acid	7.64 d (*J* = 16.0), 6.38 d (*J* = 16.0)	HSQC	2
Phenylalanine	7.37 m	HSQC	2
Chicoric acid	7.71 d (*J* = 17.0), 6.47 d (*J* = 17.0), 7.23 s, 6.90 d (*J* = 8.0)	HSQC	2
Trigonelline	9.13 s, 8.07 t	HSQC	2

^a^ MSI level of identification based on Sumner et al., 2007 [42].

## Data Availability

Data are contained within the article.
